# Age‐dependent nuclear lipid droplet deposition is a cellular hallmark of aging in *Caenorhabditis elegans*

**DOI:** 10.1111/acel.13788

**Published:** 2023-01-31

**Authors:** Konstantinos Palikaras, Meropi Mari, Christina Ploumi, Andrea Princz, George Filippidis, Nektarios Tavernarakis

**Affiliations:** ^1^ Department of Physiology, Medical School National and Kapodistrian University of Athens Athens Greece; ^2^ Institute of Electronic Structure and Laser, Foundation for Research and Technology Heraklion Greece; ^3^ Institute of Molecular Biology and Biotechnology Foundation for Research and Technology Heraklion Greece; ^4^ Medical School University of Crete Heraklion Greece

**Keywords:** aging, ATGL‐1, HLH‐30/TFEB, lipid droplet, non‐linear optical phenomena, nucleus

## Abstract

Aging is the major risk factor for several life‐threatening pathologies and impairs the function of multiple cellular compartments and organelles. Age‐dependent deterioration of nuclear morphology is a common feature in evolutionarily divergent organisms. Lipid droplets have been shown to localize in most nuclear compartments, where they impinge on genome architecture and integrity. However, the significance of progressive nuclear lipid accumulation and its impact on organismal homeostasis remain obscure. Here, we implement non‐linear imaging modalities to monitor and quantify age‐dependent nuclear lipid deposition in *Caenorhabditis elegans.* We find that lipid droplets increasingly accumulate in the nuclear envelope, during aging. Longevity‐promoting interventions, such as low insulin signaling and caloric restriction, abolish the rate of nuclear lipid accrual and decrease the size of lipid droplets. Suppression of lipotoxic lipid accumulation in hypodermal and intestinal nuclei is dependent on the transcription factor HLH‐30/TFEB and the triglyceride lipase ATGL‐1. HLH‐30 regulates the expression of ATGL‐1 to reduce nuclear lipid droplet abundance in response to lifespan‐extending conditions. Notably, ATGL‐1 localizes to the nuclear envelope and moderates lipid content in long‐lived mutant nematodes during aging. Our findings indicate that the reduced ATGL‐1 activity leads to excessive nuclear lipid accumulation, perturbing nuclear homeostasis and undermining organismal physiology, during aging.

## Graphical abstract

Age‐dependent deterioration of lipid metabolism and nuclear morphology are common features in evolutionary divergent organisms. Nuclear lipid droplet (nLDs) deposition and LMN‐1/LMNA accumulation lead to cellular dysfunction and, subsequently, to tissue homeostasis collapse, with age. Longevity‐promoting interventions sustain nLDs abundance and nuclear envelope integrity, via distinct, HLH‐30‐driven mechanisms, to protect cellular viability and promote organismal fitness.

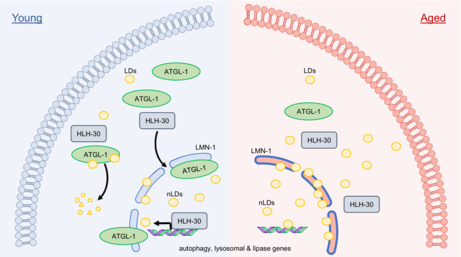

**Abbreviations**
LDs: lipid droplets
nLDs: nuclear lipid droplets
SHG: second harmonic generation
THG: third harmonic generation
TPEF: two‐photon excited fluorescence

## 1 INTRODUCTION

Lipid droplets (LDs), the major form of fat storage in cells, are evolutionarily conserved intracellular organelles, which provide a pool of energy resources and building blocks for membranes synthesis and maintenance (Farese Jr. & Walther, 2009; Kuhnlein, 2012). The cellular functions of LDs include energy storage, protection against excess lipid toxicity, and serving as a site for sequestration and degradation of targeted proteins (Farese Jr. & Walther, 2009). Lipid droplets are mainly generated from ER membranes and are localized in the cytoplasm close to the outer nuclear membrane. However, accumulating evidence demonstrates the presence of LDs inside the nucleus. Thus, the association between lipid droplets and the nucleus is an emerging topic.

Although, nuclear lipid droplets (nLD) have been described in yeast cells, hepatocytes, and more recently in osteosarcoma cells (U2OS), their cellular function remains obscure (Chin et al., 2020; Ohsaki et al., 2016; Romanauska & Kohler, 2018; Soltysik et al., 2021). Nuclear lipid droplets appear to be smaller and have different lipid composition than their cytoplasmic counterparts. Moreover, when cells are cultured in a fatty acid‐rich media, the number of nLDs is increased (Barbosa & Siniossoglou, 2020). Regarding their function, it is suggested that nLDs can serve as storage sites for histones, bind to transcription factors or regulate the transport of specific proteins between the nucleus and cytoplasm (Welte, 2015). A very recent study in *C. elegans* underlines the essential role of nLDs formation in the maintenance of intestinal and gonadal homeostasis (Mosquera et al., 2021). The enhanced number and increased size of nLDs in several human pathologies, such as atherosclerosis, obesity, fatty liver disease, and hepatic steatosis, underlines the detrimental effects of nLDs deregulation on cellular and tissue physiology (Barbosa & Siniossoglou, 2020; Sołtysik et al., 2019).

Despite the recent advances in nuclear lipid droplet research, the regulatory mechanisms dictating nuclear homeostasis collapse and nuclear lipids deposition during aging remain elusive. Here we report the use of non‐linear imaging modalities to monitor and quantify nLDs and delineate the precise connection between nuclear impairment and aging. Our studies suggest that lipid content progressively accumulates with age in the nuclear envelope in several tissues, such as hypodermis and intestine of *C. elegans*. Importantly, genetic interventions known to delay aging in different model organisms, such as low insulin signaling and dietary restriction, decrease the number of nuclear lipids and reduce their size. HLH‐30, the homolog of the mammalian TFEB and the master regulator of lysosomal function, is required for the maintenance of nLDs homeostasis. Although autophagy is a well‐known moderator of lipid content, it does not affect the age‐dependent accrual of nLDs suggesting that HLH‐30 regulates nLDs in an autophagy‐independent manner. HLH‐30 dictates the expression of several lipases, such as LIPL‐4 and ATGL‐1 (the homolog of the mammalian adipose triglyceride lipase (ATGL)). Interestingly, ATGL‐1 is located both in cytoplasm and nucleus and regulates nuclear lipid deposition in long‐lived animals. Notably, age‐dependent elevation of nuclear fat deposition is linked to increased LMN‐1/LMNA protein levels. Although nLDs and LMN‐1 abundance are governed by HLH‐30, ATGL‐1 does not influence the protein levels of LMN‐1 indicating that the regulation of these two phenomena is mediated by distinct HLH‐30‐driven mechanisms. Our findings highlight the pivotal role of HLH‐30/ATGL‐1 axis in restraining lipid expansion in the nucleus, thereby preserving nuclear lipid homeostasis and organismal fitness during aging.

## 2 RESULTS

### 2.1 Age‐dependent alterations in nuclear structure and lipid content

**FIGURE 1:**
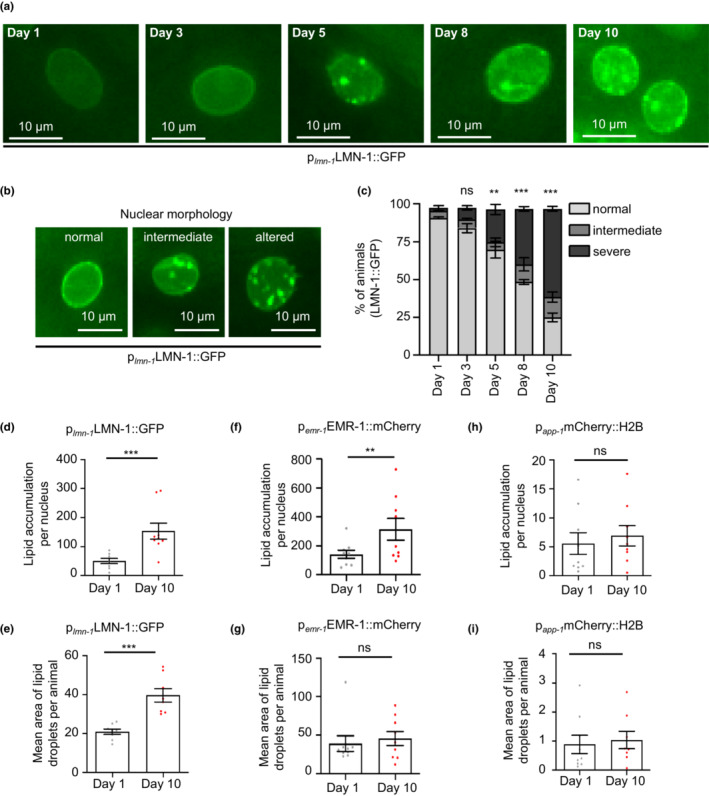
Nuclear structure is altered during aging. (a) Representative images of hypodermal nuclei in animals of the LW697 [p_*lmn‐1*_LMN‐1::GFP] reporter strain during aging. (b) Representative images depicting alterations (normal, intermediate, and severe) in the nuclear morphology of hypodermal cells in animals of the LW697 [p_*lmn‐1*_LMN‐1::GFP] reporter strain. Scale bars, 10 μm. (c) Qualitative analysis of alterations in the nuclear morphology of hypodermal cells in animals of the LW697 [p_*lmn‐1*_LMN‐1::GFP] reporter strain during aging (*n* = 50 nuclei per condition; ns *p >* 0.05, **p < 0.01, ***p < 0.001, one‐way ANOVA followed by Tukey HSD post hoc test). (d–i) Quantification of LD number and size in distinct nuclear compartments of intestinal nuclei in 1‐day‐ and 10‐day‐old animals, using a combined THG and TPEF imaging modalities. LW697 [p_*lmn‐1*_LMN‐1::GFP] and BN147 [p_*emr‐1*_EMR‐1::mCherry] reporter strains were used to identify alterations in the nuclear envelope, while the YQ243 [p_*app‐1*_H2B::mCherry] reporter strain was used to identify alterations in the intra‐nuclear space (*n* = 15 for each genetic background and time point; ns *p >* 0.05, **p < 0.01, ***p < 0.001, unpaired *t*‐test). Error bars denote the standard error of the mean.

Several hallmarks of aging are tightly associated with nuclear homeostasis (Hou et al., 2019; Lopez‐Otin et al., 2013; Schmauck‐Medina et al., 2022). Thus, it is highly appreciated that nucleus serves a paramount role in the determination of organismal healthspan and lifespan. During aging, the nucleus undergoes a functional decline that is coupled with a progressive deterioration in its morphology (Burke & Stewart, 2014; Haithcock et al., 2005; Papsdorf & Brunet, 2019; Pathak et al., 2021; Schumacher & Vijg, 2019). To examine nuclear morphology during the aging process, we utilized transgenic nematodes expressing the nuclear lamina protein LMN‐1 (the ortholog of mammalian LMNA) fused to GFP under its endogenous promoter. Several tissues, including hypodermis, body wall muscles, and intestine, showed gradual deterioration of the well‐structured nuclear shape with age (Figure 1a–c; Haithcock et al., 2005; Pathak et al., 2021). Although recent studies highlight the detrimental effects of age‐dependent and excessive deposition of cytosolic LDs both in adipose and non‐adipose tissues on organismal physiology, the abundance of nLDs and their impact on cellular function and tissue homeostasis are still obscure (Barbosa & Siniossoglou, 2020).

We utilized third harmonic generation (THG) imaging technique to detect and quantify nLDs deposition in nematodes. Third harmonic generation imaging constitutes a powerful, label‐free and non‐invasive method for the precise identification of fat content *in vivo* at microscopic level. Indeed, THG has already been used to detect lipid deposition in various biological samples including nematodes and mammalian cells (Débarre et al., 2006; Palikaras et al., 2017; Tserevelakis et al., 2014). The visualization and quantification of lipid droplets in the nuclei of *C. elegans* includes the combination of THG and two‐photon‐excitation fluorescence (TPEF) (Figure S1). Simultaneous THG and TPEF 3D imaging of 1‐ and 10‐day‐old transgenic nematodes expressing fluorescent reporters of the nuclear membrane (LMN‐1::GFP and EMR‐1::mCherry) and the nucleoplasm (H2B::mCherry) showed the presence of nLDs in the respective nuclear compartments (Figure S2A–C). To measure the levels of nLDs, we performed the following image analysis. Nuclei were manually selected and isolated from each slice of the respective image. A constant threshold was set for all the respective regions of interest (ROIs) for each image. The total sum of the LDs for each animal was calculated through the resulting binary images and the total mean areas (in pixels) were divided by the number of nuclei detected in each animal. Then, the representative index of the total nuclear lipid content within the examined part was depicted in a graph bar (Figure S3A, B). Our analysis suggests that nLDs number and size gradually increase in the nematode intestinal cells during aging. Interestingly, age‐dependent nuclear lipid deposition is prominent in the nuclear envelope (LMN‐1::GFP, EMR‐1::mCherry), whereas in the nucleoplasm (H2B::mCherry) no significant changes are observed in either nLD abundance or size (Figure 1e). Taken together, these findings established non‐linear phenomena as a novel, non‐destructive, and label‐free microscopy method to assess nLDs in vivo and underline the age‐dependent accrual of nLDs in the nuclear envelope.

### 2.2 Caloric restriction and low insulin signaling diminish nuclear lipid droplet accrual with age

Aging is a universal phenomenon that is characterized by progressive decline of cellular function and tissue homeostasis collapse leading eventually to organismal degeneration and death. Nevertheless, several environmental and genetic factors have been shown to extend lifespan through the activation of multiple molecular mechanisms, which are conserved from unicellular eukaryotic organisms to mammals (DiLoreto & Murphy, 2015; Hou et al., 2019; Kenyon, 2010; Lopez‐Otin et al., 2013; Melzer et al., 2020). Among those longevity pathways dietary restriction and low insulin signaling trigger a cascade of signaling events that stimulate subsequent metabolic changes to tackle aging and age‐related pathologies (DiLoreto & Murphy, 2015; Kenyon, 2010; Papsdorf & Brunet, 2019).

**FIGURE 2:**
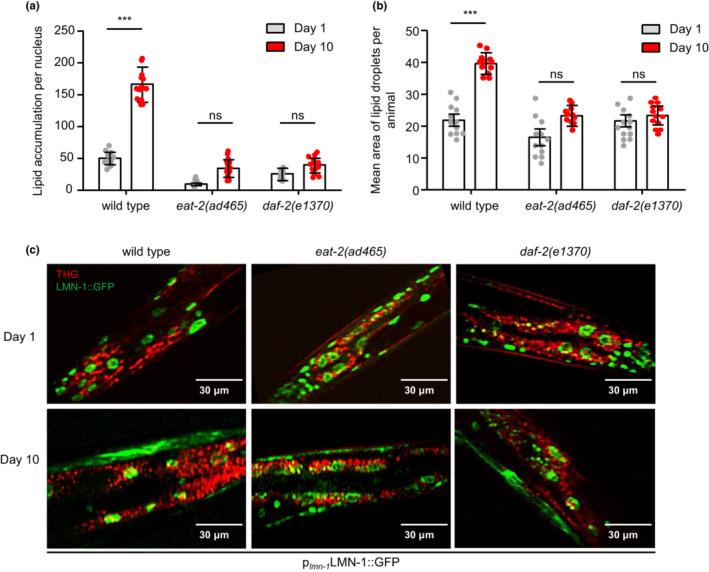
Longevity‐promoting interventions abolish the gradual nLDs accumulation. (a, b) Quantification of LDs volume per nucleus (a) and average size (b) in the intestinal nuclei of 1‐day‐ and 10‐day‐old wild‐type, *eat‐2(ad465)* and *daf‐2(e1370)* transgenic nematodes expressing LMN‐1::GFP (*n* = 15 animals for each genetic background and time point, ns *p >* 0.05, ***p < 0.001, one‐way ANOVA followed by Tukey HSD post hoc test). (c) Representative images of combined THG (red) and TPEF (green) imaging method, demonstrating LD deposition in the intestinal nuclei of 1‐day‐ and 10‐day‐old wild‐type, *eat‐2(ad465)* and *daf‐2(e1370)* transgenic nematodes expressing LMN‐1::GFP. Scale bars, 30 μm. Error bars denote standard error of the mean.

Previous studies have shown the protective effect of caloric restriction and low insulin signaling against age‐dependent ectopic fat deposition and lipotoxicity (Huffman & Barzilai, 2009; Muzumdar et al., 2008; Palikaras et al., 2017). Consistent with the previous reports, the long‐lived *eat‐2(ad465)* and *daf‐2(e1370)* mutant nematodes displayed low levels of nuclear lipids and a concomitant decrease in their size, compared to their respective wild‐type counterparts during aging (Figure 2a–c). Moreover, the quantity of nLDs is reduced in wild‐type nematodes, which are subjected to 6 h starvation during their development (Figure S4A, B). Thus, the steady levels of nLDs throughout the lifespan of long‐lived nematodes suggest that lipid metabolism is enhanced in the nuclear envelope compartments of *eat‐2(ad465)* and *daf‐2(e1370)* mutants, resulting in less and smaller nLDs.

**FIGURE 3:**
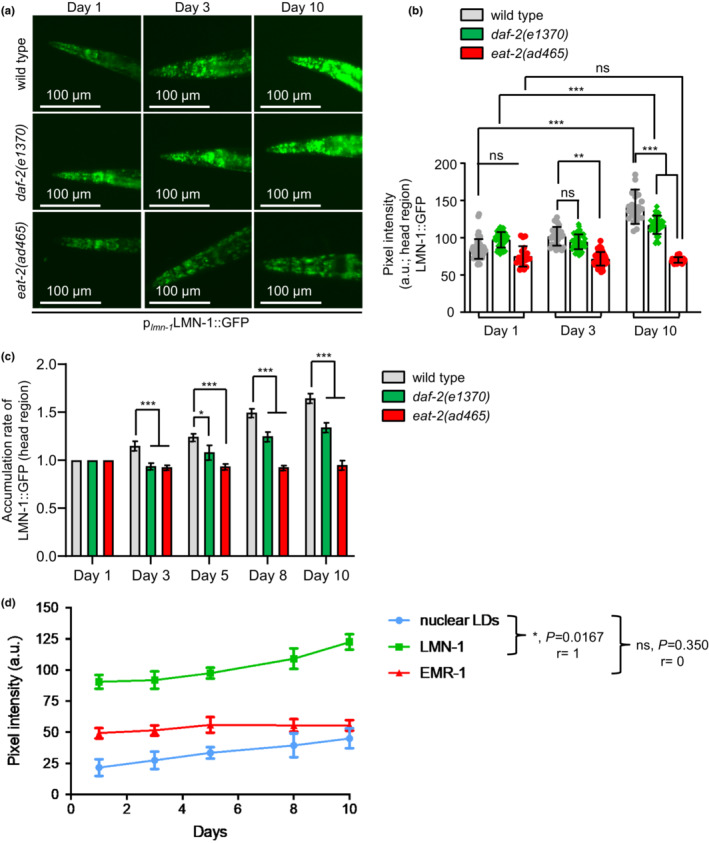
Age‐dependent accumulation of LMN‐1 protein levels. (a) Representative images of wild‐type, *daf‐2(e1370)* and *eat‐2(ad465)* transgenic nematodes expressing LMN‐1::GFP during aging. Images were acquired using an X5 objective lens. Scale bars, 100 μm. (b) Quantification analysis of the mean fluorescence intensity in the head region of wild‐type, *daf‐2(e1370)*, and *eat‐2(ad465)* transgenic nematodes expressing LMN‐1::GFP during aging (*n* = 50 animals for each genetic background and time point, ns *p >* 0.05, **p < 0.01, ***p < 0.001, one‐way ANOVA followed by Tukey HSD post hoc test). (c) Accumulation rate of LMN‐1::GFP in the head region of wild‐type, *daf‐2(e1370)* and *eat‐2(ad465)* transgenic nematodes during aging (*n* = 50 animals for each genetic background and time point, *p < 0.05, ***p < 0.001, one‐way ANOVA followed by Tukey HSD post hoc test). (d) Correlation between the number of nLDs and the protein levels of LMN‐1::GFP and EMR‐1::mCherry during aging (ns *p >* 0.1234, *p < 0.03; non‐parametric Spearman correlation, *r* = 1). Error bars denote standard error of the mean.

Nuclear envelope is a membranous and multiprotein structure that separates the nuclear from cytosolic compartments in eukaryotic cells. A wide variety of cellular processes, including gene expression, DNA metabolism, and chromatin organization among others, are coordinated by the dynamic nature of nuclear envelope, highlighting its essential role for cellular homeostasis (Cohen‐Fix & Askjaer, 2017; Hetzer, 2010). Regarding its architecture, nuclear envelope is composed of three main domains: the outer nuclear membrane, the inner nuclear membrane, and the nuclear lamina (Cohen‐Fix & Askjaer, 2017; Hetzer, 2010). A recent study in *Saccharomyces cerevisiae* uncovered that inner nuclear membrane is a metabolic active region, where local lipid metabolism is taking place to regulate transcription and lipid homeostasis (Romanauska & Kohler, 2018). To examine the impact of aging on nuclear envelope domains, we used transgenic nematodes expressing the inner nuclear membrane protein EMR‐1 fused to mCherry (the homolog of the mammalian emerin) and the nuclear lamina protein LMN‐1 fused with to GFP. We found that the abundance of LMN‐1 is gradually elevated with age, whereas the protein levels of EMR‐1 are not altered (Figure 3a–c and Figure S5A–E). Interestingly, this differential effect of aging on LMN‐1 and EMR‐1 protein levels is highly correlated with nLDs accumulation, indicating an intricate association between LMN‐1 and nLDs (Figure 3d). Further supporting this notion, the protein levels and the accumulation rate of EMR‐1 remain stable in the long‐lived nematodes, whereas LMN‐1 levels are stable in *eat‐2(ad465)* and display a slower accumulation rate in DAF‐2 deficient animals during aging (Figure 3a–c and Figure S5A–E). Collectively the above results suggest that both caloric restriction and low insulin signaling regulate the abundance of LMN‐1 protein and nLDs sustaining nuclear lipid homeostasis.

Vitellogenins are highly expressed and large proteome contributors influencing intestinal lipid localization and distribution in *C. elegans* during aging. Notably, vitellogenins production is particularly reduced in long‐lived mutant nematodes, including *eat‐2(ad465)* and *daf‐2(e1370)* (Dong et al., 2007; Murphy et al., 2003). Accumulating evidence suggests that VIT‐2 protein levels are increased during aging and its depletion extends lifespan in *C. elegans*, while the expression of *vit‐2* gene is suppressed in *daf‐2(e1370)* long‐lived animals (DePina et al., 2011; Goszczynski et al., 2016; Mallick & Gupta, 2020; Murphy et al., 2003). Given that vitellogenins' production in the ER continues beyond the reproductive stage, it can be speculated that nLDs may arise from mis‐regulated vitellogenin production. Interestingly, knocking down of *vit‐2* gene does not abolish nLDs' abundance during aging (Figure S6A, B). Though, it seems that deficiency in VIT‐2 expands nLD size in both young and old ages (Figure S6B). Moreover, VIT‐2 depletion does not affect the levels of LMN‐1::GFP in wild‐type and *eat‐2(ad465)* nematodes, while it increases LMN‐1::GFP fluorescent signal in *daf‐2(e1370)* mutants (Figure S6C). Taken together, these findings indicate that different longevity interventions differentially modify cytosolic and nuclear lipid droplets accumulation, though further investigation is needed.

### 2.3 HLH‐30 and ATGL‐1 regulates nuclear lipid deposition in long‐lived animals

**FIGURE 4:**
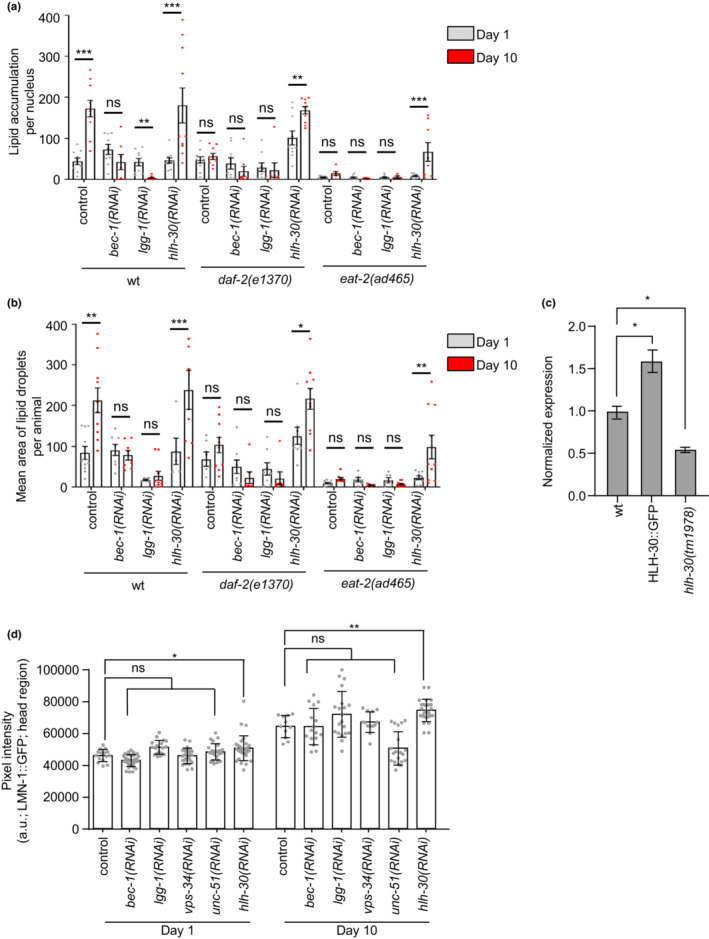
HLH‐30 regulates nLDs deposition in wt and long‐lived mutants. (a, b) Quantification of LDs volume per nucleus (a) and average size (b) in the intestinal nuclei of 1‐day‐ and 10‐day‐old wild‐type, *daf‐2(e1370)* and *eat‐2(ad465)* transgenic nematodes expressing LMN‐1::GFP. The animals were treated with empty vector (control), *bec‐1(RNAi)*, *lgg‐1(RNAi)*, and *hlh‐30(RNAi)* (*n* = 15 animals for each genetic background and time point, ns *p >* 0.05, *p < 0.05, **p < 0.01, ***p < 0.001, one‐way ANOVA followed by Tukey HSD post hoc test). (c) Quantification of *atgl‐1* mRNA levels in 1‐day‐old animals of: wild‐type, MAH240: *sqIs17*[p_*hlh‐30*_:HLH‐30::GFP+ *rol‐6(su1006)*], and *hlh‐30(tm1978)* strains. mRNA expression levels are relative to control and are normalized to the reference gene *pmp‐3* (*n* = 3, *p < 0.05, one‐way ANOVA/Tukey's multiple comparison test). (d) Quantification analysis of the mean fluorescence intensity in the head region of 1‐day‐ and 10‐day‐old wild‐type transgenic nematodes expressing LMN‐1::GFP. The animals were treated with empty vector (control), *bec‐1(RNAi)*, *lgg‐1(RNAi)*, *vps‐34(RNAi)*, *unc‐51(RNAi)*, and *hlh‐30(RNAi)* (*n* = 15 animals for each genetic background and time point, ns *p >* 0.05, *p < 0.05, **p < 0.01, one‐way ANOVA followed by Tukey HSD post hoc test). Error bars denote standard error of the mean.

Emerging findings suggest that the catabolic cellular process of autophagy regulates lipid metabolism through the direct degradation of LDs in several cell types (Aman et al., 2021; Klionsky et al., 2021; Liu & Czaja, 2013; Mizushima & Levine, 2020). It is widely appreciated that autophagy efficiency declines with age leading to excessive accrual of damaged organelles, toxic protein aggregates and subsequently to cellular and tissue degeneration (Aman et al., 2021; Klionsky et al., 2021; Mizushima & Levine, 2020). To elucidate whether the age‐dependent accumulation of nLDs depends on autophagy, we monitored nLDs upon depletion of the autophagy‐related factors BEC‐1 and LGG‐1. Notably, BEC‐1 and LGG‐1 deficiency (the homolog of the mammalian Beclin and GABARAP respectively) does not affect either the number or the size of nLDs in 1‐ and 10‐day‐old wild‐type, *daf‐2(e1370)*, and *eat‐2(ad465)* nematodes (Figure 4a, b). Moreover, deficiency of LGG‐2, the homolog of the mammalian LC3 autophagosomal protein, does not influence the quantity and size of nLDs both in wild‐type and *daf‐2(e1370)* animals (Figure S7A–C). Notably, efficient autophagic machinery regulates the storage and the distribution of general lipid content during nematode development (Lapierre, Silvestrini, et al., 2013). However, the developmental impact of autophagy on nLDs homeostasis cannot be discriminated by its cytoprotective function in our experimental setup. Thus, further studies should focus on this direction.

HLH‐30 is the master regulator of lysosomal function and autophagy through the expression of several autophagy genes and lysosomal lipases in response to lifespan‐extending conditions (Lapierre, De Magalhaes Filho, et al., 2013; O'Rourke & Ruvkun, 2013; Silvestrini et al., 2018). Interestingly, HLH‐30 depletion increased the abundance of nLDs (Figure 4a, b). Our findings clearly display that age‐dependent increase in nLDs number and size is not mediated by autophagic pathway impairment (Figure 4a, b). Thus, we examined the role of cytosolic lipolytic enzymes that are implicated in the regulation of LDs abundance and morphology under normal and challenged conditions (Grabner et al., 2021). Intriguingly, knocking down the longevity‐promoting lysosomal lipase LIPL‐4, a well‐known target of HLH‐30, abolished the elevated nLDs levels in wild‐type nematodes during aging (Figure S8A, B) (Folick et al., 2015; Ramachandran et al., 2019). This finding indicates that LIPL‐4 activity is required for the generation of nLDs and does not act in the same pathway with HLH‐30 to modulate age‐dependent nuclear lipid content accrual.

**FIGURE 5:**
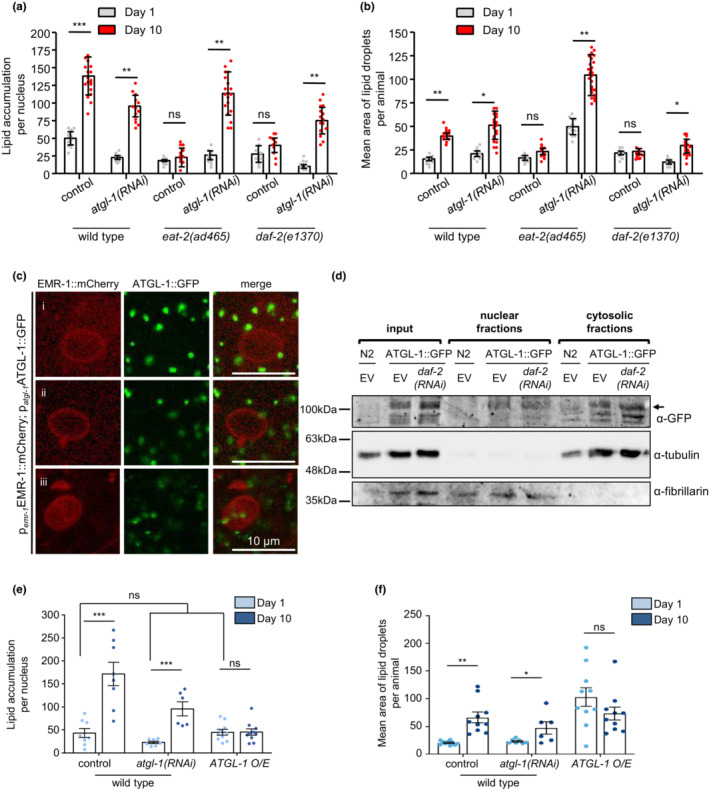
The triglyceride lipase ATGL‐1 sustains nuclear lipid content in long‐lived nematodes. (a, b) Quantification of LDs volume per nucleus (a) and average size (b) in the intestinal nuclei of wild‐type, *eat‐2(ad465)* and *daf‐2(e1370)* nematodes expressing LMN‐1::GFP. The animals were treated with empty vector (control) and *atgl‐1(RNAi)* (*n* = 15 animals for each genetic background and time point, ns *p >* 0.05, *p < 0.05, **p < 0.01, ***p < 0.001, one‐way ANOVA followed by Tukey HSD post hoc test). (c) Representative images of transgenic animals co‐expressing a full‐length p_*atgl‐1*_ATGL::GFP reporter fusion (VS20 strain) together with the nuclear envelope reporter p_*emr‐1*_EMR‐1::mCherry. Scale bars, 10 μm. (d) Western blot analysis for the detection of ATGL‐1::GFP in nuclear and cytosolic fragments of the VS20 [p_*atgl‐1*_ATGL::GFP] nematode strain. The animals were treated with empty vector (EV) and *daf‐2(RNAi)*. Fibrillarin and tubulin were used as loading controls for nuclear and cytosolic fractions respectively. Lysates from wild‐type animals were used as controls to verify that α‐GFP specifically detects ATGL‐1::GFP. (e, f) Quantification of LDs volume per nucleus (e) and average size (f) in the intestinal nuclei of 1‐day‐ and 10‐day‐old LMN‐1::GFP transgenic wild‐type animals, treated with empty vector (control) or *atgl‐1(RNAi)* and ATGL‐1 overexpressing animals (IR3160 line), treated with empty vector (control) (*n* = 15 animals for each genetic background and time point, ns *p >* 0.05, *p < 0.05, **p < 0.01, ***p < 0.001, one‐way ANOVA followed by Tukey HSD post hoc test). Error bars denote standard error of the mean.

Recent evidence supports that the lifespan‐extending properties of dietary restriction and low insulin signaling depend on the rate‐limiting lipolytic enzyme ATGL‐1, the homolog of the mammalian adipose triglyceride lipase (Zaarur et al., 2019) (Figure S9C). Interestingly, the fact that ATGL‐1 is transcriptionally regulated by HLH‐30 highlights their tight co‐regulation in response to longevity‐promoting interventions (Figure 4c) (Lapierre, De Magalhaes Filho, et al., 2013; O'Rourke & Ruvkun, 2013; Zaarur et al., 2019). Indeed, ATGL‐1 deficiency results in elevated lipid content and lifespan shortening in *eat‐2(ad465)* and *daf‐2(e1370)* mutants (Zaarur et al., 2019). Although knocking down of ATGL‐1 did not influence nLDs deposition in young wild‐type and mutant nematodes, its depletion resulted in elevated nLDs number in aged nematodes (Figure 5a, e and Figure S9A). Moreover, ATGL‐1 deficiency mediated the enlargement of nLDs shape in the intestinal cells of old wild‐type, *eat‐2(ad465)*, and *daf‐2(e1370)* animals (Figure 5b). Although ATGL‐1 is reported as a cytosolic lipase, *in silico* analysis identified a nuclear localization sequence (NLS; Figure S9B) close to the N‐terminal of the protein (Kosugi et al., 2009). To validate the cellular localization of ATGL‐1, we generated transgenic nematodes expressing ATGL‐1::GFP together with the inner nuclear membrane marker EMR‐1::mCherry. In addition to its widely cytoplasmic distribution, ATGL‐1::GFP is found to be in proximity to the nuclear envelope (Figure 5c‐i), be co‐localized with EMR‐1::mCherry (Figure 5c‐ii) and in the nucleoplasm (Figure 5c‐iii). Furthermore, biochemical analysis shows that ATGL‐1::GFP is detected both in cytosolic and nuclear extracts (Figure 5d). Interestingly, ATGL‐1::GFP levels are slightly elevated in the nuclear extracts of the long‐lived *daf‐2(e1370)* nematodes further supporting its essential role in nLDs regulation (Figure 5d). To examine whether ATGL‐1 activity is sufficient for the maintenance of nLDs, we generated transgenic nematodes overexpressing ATGL‐1, without any fusion tag, under its endogenous promoter (Figure S9D). We found that ATGL‐1 overexpressing animals display diminished nLD accumulation during aging (Figure 5e, f).

To investigate further the molecular mechanism that dictates the tight association between nLDs and LMN‐1 accrual with age, we monitored the LMN‐1::GFP signal in ATGL‐1 overexpressing nematodes. ATGL‐1 overexpression does not affect either the basal protein levels or the age‐dependent accumulation of LMN‐1 (Figure S9E). Moreover, the nuclear morphology deterioration is not rescued upon ATGL‐1 up‐regulation (Figure S9F). Although, LMN‐1::GFP levels are not altered upon depletion of the core component of general autophagic machinery, such as LGG‐1, BEC‐1, VSP‐34, and UNC‐51, HLH‐30 deficiency results in elevated LMN‐1::GFP levels, suggesting that LMN‐1 is degraded via a distinct HLH‐30‐driven mechanism that is independent of autophagy and ATGL‐1 function (Figure 4d).

## 3 DISCUSSION

During the last decade, emerging findings have associated nuclear envelope impairment with the development and progression of several pathological conditions (Pathak et al., 2021; Schumacher & Vijg, 2019; Stewart et al., 2007). Lipid metabolism interferes with the balanced nuclear function and integrity, highlighting its essential role in cellular and tissue homeostasis (Barbosa & Siniossoglou, 2020; Mosquera et al., 2021; Papsdorf & Brunet, 2019; Romanauska & Kohler, 2018; Welte, 2015; Zamboni et al., 2014). The association between LDs and the nucleus is not surprising since de novo synthesis of LDs is taking place in the ER, and the ER is contiguous with the nuclear envelope. Although LDs have been shown to surround the nucleus in various cell types, their presence inside the nuclear compartments was recently observed (Barbosa & Siniossoglou, 2020; Mosquera et al., 2021; Ohsaki et al., 2016; Soltysik et al., 2021; Sołtysik et al., 2019). In this study, we combined non‐destructive and label‐free non‐linear imaging modalities to monitor and quantify nLDs. Although nuclear fat was detected in the nuclear compartments of hypodermal and intestinal cells, we found that nLDs were progressively accumulated only in the nuclear envelope during aging. These findings agree with recent studies in yeast and mammalian cells showing that inner nuclear membrane is a metabolically active area in which nLDs are synthesized (Romanauska & Kohler, 2018; Soltysik et al., 2021). A very recent study in *C. elegans* demonstrated that nuclear fat is detected in intestinal and germ cells regulating multiple aspects of their physiology (Mosquera et al., 2021). However, the function of nLDs in nuclear integrity and homeostasis, as well as the metabolic pathways that regulate nLDs size and abundance remain elusive.

Consistent with accumulating evidence that caloric restriction and low insulin signaling extend lifespan across species, and delay the age‐dependent abnormalities of nuclear morphology, we found that *eat‐2(ad465)* and *daf‐2(e1370)* mutant nematodes maintain the number and the size of nLDs in their intestinal cells during aging (Charar et al., 2021; Haithcock et al., 2005; Pathak et al., 2021; Perez‐Jimenez et al., 2014). Intriguingly, the overall lipid content is shown to be elevated in the long‐lived *daf‐2(e1370)* mutant nematodes (Lapierre, Silvestrini, et al., 2013; O'Rourke et al., 2009). These findings suggest that nLDs homeostasis is differentially regulated uncoupling their levels from total lipid content. Investigating the effect of aging on nuclear envelope domains, we found that the levels of the inner nuclear membrane protein EMR‐1 are not changed, whereas the nuclear lamina protein LMN‐1 is increased in aged nematodes. The pivotal role of nuclear envelope domains in nLDs homeostasis is further demonstrated by the fact that age‐associated nLDs elevation is positively correlated with LMN‐1 protein levels. Notably, *eat‐2(ad465)* and *daf‐2(e1370)* long‐lived mutants display stable LMN‐1 abundance underlining that both longevity interventions regulate the nuclear lamina components to sustain nLDs homeostasis during aging. Interestingly, mutations in genes that encode nuclear lamina factors, such as LMNA, are linked to human aging and progeria syndromes and their malfunction results in a multitude of diseases, including muscle dystrophies and lipodystrophies (de Leeuw et al., 2018; Scaffidi & Misteli, 2006). A recent study unraveled that missense mutations in *LMNA* gene result in altered transcriptional program of metabolic‐related genes and irregular nuclear shape in human patients (Desgrouas et al., 2020). Furthermore, human cells derived from metabolic syndrome patients display abnormal nuclear morphology and impaired nuclear distribution of lamin A/C (Dutour et al., 2011). These findings further support the existence of a tight coordination between the nuclear lamina and nLDs that influences cellular and tissue metabolism. However, further investigation is required towards this direction, since nLDs distribution in nuclear compartments has not been examined in cells derived from either progeria syndrome or metabolic syndrome patients.

Besides the physical association of LDs with the nuclear compartments, little is known about their functional role in nuclear homeostasis. It is suggested that LDs modulate nuclear integrity by storing histones, binding to transcription factors, and physically interacting with other proteins to regulate their transport between cytoplasm and nucleoplasm (Barbosa & Siniossoglou, 2020; Sołtysik et al., 2019; Welte, 2015). Indeed, genetic studies in flies have shown that Jabba, an LD surface protein, interacts with histones mediating their storage during embryo development (Cermelli et al., 2006). Despite their role as transient storage depots, LDs could also supply histones for rapid chromatin remodeling (Li et al., 2012, 2014). Additionally, nLDs have the potential to influence chromatin organization since they are formed at the envelope and, thereby, penetrating nuclear lamina to enter the nucleoplasm (Mosquera et al., 2021; Ohsaki et al., 2016; Romanauska & Kohler, 2018). A very recent study conducted in *C. elegans* reported that nLDs are coated by LMN‐1 and/or heterochromatin, suggesting that nLDs accumulation could mediate the disposal of peripheral heterochromatin (Mosquera et al., 2021). Interestingly, loss of heterochromatin and derepression of several silenced genes are highly appreciated as major contributors to premature aging (Tsurumi & Li, 2012). Moreover, excessive accrual of nLDs could disrupt chromosome territories leading eventually to nuclear damage and cellular dysfunction. Indeed, the loss of the intestinal nuclei is a well‐characterized feature of *C. elegans* aging that could be driven by the runaway accumulation of nLDs (Gems & Riddle, 2000; Mosquera et al., 2021; Poteryaev et al., 2005).

Genetic studies in *C. elegans* demonstrated that functional autophagy during early developmental stages is required for lipid droplet homeostasis (Lapierre, Silvestrini, et al., 2013). Indeed, BEC‐1, LGG‐1, UNC‐51, and VSP‐34 depleted nematodes display reduced general lipid content both in wild‐type and long‐lived *daf‐2(e1370)* nematodes, highlighting the essential developmental role of autophagy in lipid storage and distribution (Lapierre, Silvestrini, et al., 2013). Although we knocked down several autophagy‐related factors from early development, we did not observe any alteration in nuclear morphology, LMN‐1::GFP abundance and nLDs number and size, suggesting that nLDs storage can be differentially regulated from cytosolic LDs. However, further investigation is needed to discriminate between the developmental and cytoprotective functions of autophagy, focusing on its impact in nLDs regulation.

Accumulating evidence suggests that HLH‐30/TFEB is a crucial regulator of autophagy, lysosomal and lysosomal lipases gene expression. Our findings clearly demonstrate that HLH‐30 is required for the reduced accrual of nLDs in long‐lived nematodes. The lysosomal lipase LIPL‐4 is highly appreciated as a longevity modulator (Folick et al., 2015; Ramachandran et al., 2019; Savini et al., 2022; Wang et al., 2008). LIPL‐4 is highly expressed in long‐lived mutant animals and its depletion abolishes the lifespan extension indicating its cytoprotective function (Wang et al., 2008). Even though LIPL‐4 deficiency does not influence the lifespan of wild‐type nematodes, its overexpression is sufficient to promote longevity (Folick et al., 2015). Although LIPL‐4 serves as a lipolysis mediator, its depletion abolishes nLDs accumulation with age in wild‐type animals. These findings underline the context‐dependent role of LIPL‐4 in cytosolic and nuclear fat deposition. Hence, future studies are required to examine the intricate role of LIPL‐4 activity in nLDs homeostasis and organismal physiology.

Recent studies suggest that among other lipolytic enzymes, ATGL‐1 is activated to promote longevity under low insulin signaling and dietary restriction (Lapierre, De Magalhaes Filho, et al., 2013; O'Rourke & Ruvkun, 2013; Zaarur et al., 2019). We found that ATGL‐1 is transcriptionally regulated by HLH‐30, indicating that HLH‐30/ATGL‐1 axis is triggered to maintain LDs homeostasis in nuclear compartments with age. Suppression of lipotoxic accumulation of lipids in the intestinal nuclei is dependent on the triglyceride lipase ATGL‐1 activity. Indeed, ATGL‐1 localizes to the nuclear envelope and sustains lipid content in long‐lived mutant nematodes during aging. Further supporting the pivotal role of ATGL‐1 in tissue and organismal homeostasis, ATGL‐1 overexpression is sufficient to sustain nLDs abundance during aging and promote lifespan extension in wild‐type nematodes (Zaarur et al., 2019). In addition to nematode data, ATGL (the human homolog of ATGL‐1) is detected both in LDs and the nucleoplasm of multiple human cell lines (www.proteinatlas.org), highlighting its possible function as nLDs regulator across species (Thul et al., 2017; Uhlén et al., 2015). Notably, ATGL was shown to interact directly with the autophagosomal protein LC3, suggesting an interplay between lipolysis and lipophagy (Martinez‐Lopez et al., 2016). However, LGG‐1, LGG‐2, and BEC‐1 deficiency do not display any effect in nLDs number and size both in aged wild‐type and long‐lived mutants, indicating that nuclear fat deposition is not regulated by autophagy. Accumulating evidence showed that nuclear export inhibition improves organismal proteostasis and longevity by promoting HLH‐30/TFEB nuclearization and autophagy induction in nematodes, flies, and mammalian cells (Silvestrini et al., 2018). Intriguingly, the autophagy protein LGG‐1 modulates nucleolar size and ribosomal function through the degradation of key components in nucleolar dynamic and protein translation, such as FIB‐1 and RPL‐11 to promote longevity upon nuclear export inhibition (Kumar et al., 2022; Tiku et al., 2018). These findings further support the notion that autophagy, apart from being a simple degradation pathway, it has a broader role in lipid remodeling and nuclear homeostasis (Lapierre, Silvestrini, et al., 2013).

**FIGURE 6:**
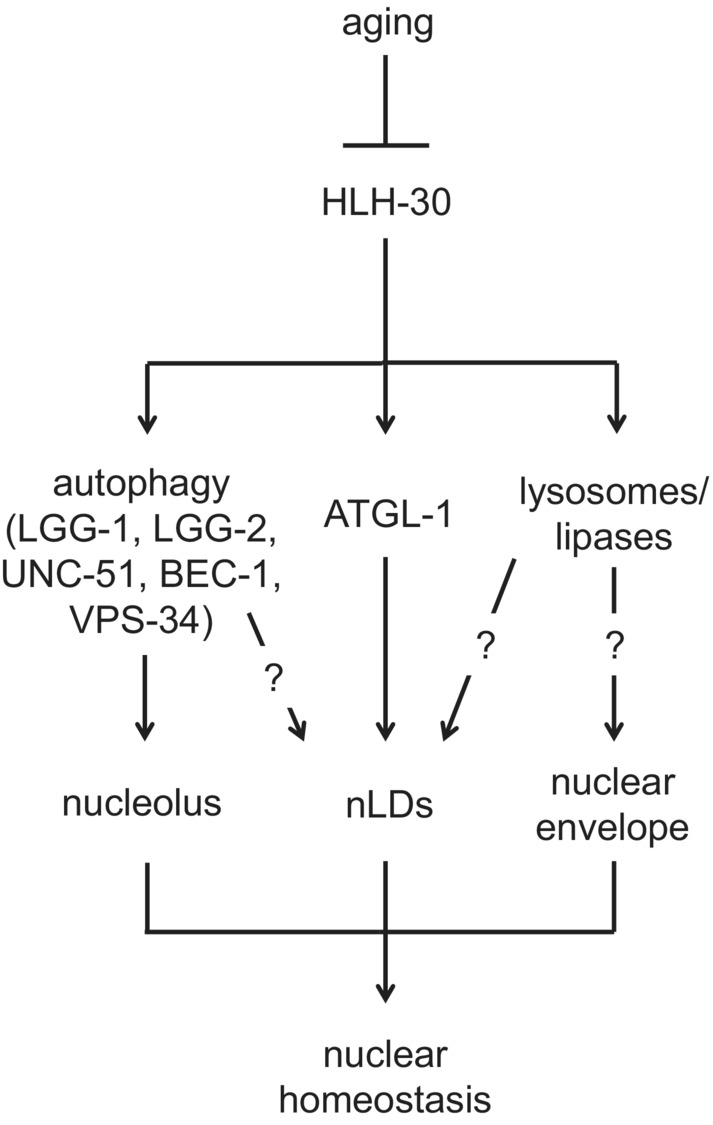
HLH‐30/ATGL‐1 axis modulates nLDs homeostasis during aging. Diminished HLH‐30 nuclearization and activity lead to excessive cellular dysfunction and subsequently to tissue homeostasis collapse with age. HLH‐30 is a central moderator of autophagy, lipolytic enzymes, such as ATGL‐1, lysosomes, and lysosomal acid lipases. Several aspects of nuclear homeostasis, including nucleolar morphology, nLDs abundance, and nuclear envelope integrity, are regulated by distinct HLH‐30‐driven mechanisms to protect cellular viability and promote organismal fitness.

The cumulative results of the current study suggest that the reduced levels and the impaired activity of HLH‐30 results in elevated LMN‐1 abundance and diminished ATLG‐1 expression, which subsequently trigger excessive nuclear lipids accumulation (Figure 6). Furthermore, our findings underscore the dynamic interplay between autophagy, lipolytic enzymes, and lipases, which is pivotal for the maintenance of nuclear envelope integrity, nucleolus morphology and nLDs content to promote nuclear homeostasis and prevent age‐dependent cellular impairment and physiology decline. *C. elegans* is a versatile genetic model that can be used as a screening platform to unravel novel tissue‐specific modulators of nLDs distribution. Investigating further the molecular pathways that regulate nLDs formation and accrual will enlighten new avenues for therapeutic intervention strategies to tackle metabolic and age‐associated diseases.

## 4 MATERIALS AND METHODS

### 4.1 Non‐linear microscopy setup

The experimental setup was similar to the one described in our previous studies (Palikaras et al., 2017; Tserevelakis et al., 2014). Two different femtosecond (fs) laser sources are employed in the framework of this study. An Yb‐based solid‐state fs laser oscillator, emitting near‐infrared pulsed light at a central wavelength of 1028 nm (t‐pulse, Amplitude systems, 200 fs, 50 MHz 1 W) is used as excitation source for the irradiation of the unstained and GFP labeled animals. A 1064 nm fiber laser (FemtoPower 1060‐5‐150‐s, 1064 nm, 80 MHz, 4 W, 150 fs, Fianium Ltd) is utilized on the same set‐up as a source for the imaging of transgenic animals expressing EMR‐1::mCherry. The laser beam is guided to a modified upright microscope (Nikon Eclipse ME600D). Adjustable neutral density filters (New Focus) are employed to control the power at the sample plane. A telescope system is utilized to control the beam radius. A set of galvanometric mirrors (Cambridge Technology) is used to perform the fast raster scanning in the selected xy plane of the *C. elegans* sample. The focal plane is adjusted by using a motorized translation stage (Standa Ltd., 1 μm minimum step). Diffraction limited focusing is achieved by using a high numerical aperture objective lens (Carl Zeiss, C‐Achroplan 32Χ, NA 0.85, water immersion). Specimens are placed between two thin (~70 μm) glass coverslips (Marienfeld). The glass slides are separated by a 100 μm spacer in order to protect the samples. The experimental apparatus allows the collection of two different non‐linear optical signals simultaneously (in the reflection and in the transmission mode). For the experiments, THG is detected in the forward path, while two‐photon excitation fluorescence (TPEF) signals are detected in the backward direction simultaneously. This quality of the optical system makes it capable of performing colocalization measurements. Sample scanning and data acquisition are controlled through a LabVIEW interface adapted to the experiment requirements. The bright field observation of nematodes is performed via a CCD camera (PixeLINK). The signals in the reflection mode (TPEF) are recorded by a photomultiplier tube (PMT; Hamamatsu) that is attached to the microscope eyepiece site and wired to the computer. For the TPEF measurements, a bandpass pass filter (525/50 Chroma) is utilized for the detection of the LMN‐1::GFP nuclei, while a bandpass filter (610 nm/10 nm, Thorlabs), is used for the detection of the TPEF arising from the EMR‐1::mCherry nuclei.

For the forward detection path (THG signals), a condenser lens (Carl Zeiss, PlanNeofluar, 40×, 0.85 NA, air immersion), a colored glass filter (U 340‐Hoya), and a second PMT (Hamamatsu) are used. The laser power on the sample plane is >1 nJ per pulse for all measurements. Our setup scans 500 × 500 pixels THG and TPEF image in 1 s. To improve the signal‐to‐noise ratio (SNR), 30 scans are averaged for each final image. To further improve image quality, a series of 2D optical sections are acquired at 2 μm intervals (*z* stack) and projected (maximum intensity projection) onto a single plane. Image J is used to process the obtained data (National Institutes of Health; http://imagej.nih.gov/ij/).

### 4.2 Imaging and statistical analysis

All the samples have been imaged under constant irradiation conditions (mean energy per pulse, linear polarization of the incident beam at the sample plane, dimensions of the scanning region, number of pixels, amplification of the PMT units). THG and TPEF intensity values collected through the PMTs are stored in 2D 500 by 500 matrices, representing each slice image of the post‐vulva region of each animal. The nuclear areas are located through the TPEF signals and are manually selected. These regions of interest (ROIs) define the lipid droplets through THG imaging on the whole volume of each nucleus. THG signal quantification is performed by setting a threshold in the obtained normalized slice images consequently, regions generating high levels of non‐linear signal (mainly corresponding to lipid particles in nucleus) are solely detected and isolated. Normalized 8‐bit slice images of the sample are processed by Image J. Thresholding follows using a constant threshold value so that only the highest THG signals are recorded and quantified. The generated stack of binary images following the thresholding procedure represents exclusively the lipid droplets in the selected nuclear regions defined by TPEF signals, while the lower THG signals arising from other inhomogeneous structures are effectively eliminated. Lipid content is measured by calculating the total area of detected regions for all sequential optical planes covering the sample depth. In addition, the same process is able to calculate the average lipid droplet size for each animal. Upon setting the same threshold on THG signals, the total lipid area as well as the number of particles are measured for each animal. The ratio of the *Total lipid Area/Number of particles* results in the average droplet size of each animal and this is another way to compare the lipid content among the different strains.

At least 15 animals have been imaged for each genetic background and age point examined. Mean pixel intensities are calculated by averaging values obtained for each image, after thresholding. Detection and quantification of the lipid droplet regions in the resulting binary images stack are performed through the *Analyze Particles* function of Image J. The total sum of the detected areas (in pixels) divided by the number of nuclei for each animal, is calculated as a representative index of the total lipid content within the examined part of post‐vulva region. Total lipid particle area and average lipid droplet size measurements of different samples are compared by one‐way ANOVA, followed by Tukey honest significant difference (HSD) post hoc tests (SPSS, IBM Corp.).

### 4.3 *Caenorhabditis elegans* strains and culture methods

We followed standard procedures for *C. elegans* strain maintenance (Stiernagle, 2006). Nematode rearing temperature was kept at 20°C unless noted otherwise. For the RNAi experiments, synchronous animal populations for each strain were generated by bleaching and the resulting eggs were placed immediately on freshly made RNAi plates, seeded with IPTG‐supplemented (2 mM) HT115 bacteria, which had already been transformed with either the empty RNAi vector pL4440 or the indicated RNAi construct. The following strains were used in this study: N2: wild‐type Bristol isolate, CB1370: *daf‐2(e1370)III*, DA465: *eat‐2(ad465)II*, *hlh‐30(tm1928)IV*, MAH240: *sqIs17*[p_*hlh‐30*_HLH‐30::GFP; *rol‐6(su1000)*], VS20: *hjIs67*[p_*atgl‐1*_ATGL‐1::GFP; p_*mec‐7*_RFP], IR2866: *hjIs67*[p_*atgl‐1*_ATGL‐1::GFP; p_*mec‐7*_RFP]; *bqSi142*[p_*emr‐1*_EMR‐1::mCherry; *unc‐119(+)*]*II*. To monitor nuclear morphology and lipid content, we used the following transgenic animals: YQ243: *wfIs232*[p_*app‐1*_mCherry::H2B; *unc‐119(+)*], LW697: *ccIs4810*[p_*lmn‐1*_LMN‐1::GFP; *dpy‐20(+)*]*I*, IR2863: *ccIs4810*[p_*lmn‐1*_LMN‐1::GFP; *dpy‐20(+)*]*I; daf‐2(e1370)III*, IR2538: *ccIs4810*[p_*lmn‐1*_LMN‐1::GFP; *dpy‐20(+)*]*I; eat‐2(ad465)II*, BN147: *emr‐1(gk119)I; bqSi142*[p_*emr‐1*_EMR‐1::mCherry; *unc‐119(+)*]*II*, IR2867: *bqSi142*[p_*emr‐1*_EMR‐1::mCherry; *unc‐119(+)*]*II; daf‐2(e1370)III*, IR2682: *eat‐2(ad465)II; bqSi142*[p_*emr‐1*_EMR‐1::mCherry; *unc‐119(+)*]*II*, IR3160: *ccIs4810*[p_*lmn‐1*_LMN‐1::GFP; *dpy‐20(+)*]*I; Ex001*[p_*atgl‐1*_ATGL‐1; *rol‐6(su1000)*].

### 4.4 Molecular cloning

For generating the ATGL‐1 O/E vector, we amplified the complete *atgl‐1* genomic sequence, including *atgl‐1* promoter region (approximately 1.5 kb upstream from the start codon) and its endogenous 3’‐UTR (approximately 400 bp downstream from the stop codon). The resulting fragment was ligated to pCRII‐TOPO and was used directly for the generation of transgenic animals.

For engineering, the *atgl‐1*, *lgg‐2*, *vit‐2*, and *lipl‐4* RNAi constructs, gene‐specific fragments of interest were obtained by PCR amplification directly from *C. elegans* genomic DNA. The PCR‐generated fragments were subcloned into the pL4440 plasmid vector. The resulting constructs were transformed into HT115(DE3) *Escherichia coli* bacteria deficient for RNase III. Bacteria carrying an empty vector were used in control experiments. The primers used for each construct are provided in Table S1. In our study we have also used RNAi vectors against *bec‐1*, *lgg‐1*, *hlh‐30*, and *vps‐34*, which had been previously generated in our lab (Samara et al., 2008). The entire list of the primers used for these genes are also included in Table S1.

### 4.5 RNA isolation and qRT‐PCR analysis

Total RNA from synchronized day‐1 adult animals was extracted by using the TRIzol reagent (Invitrogen). cDNA synthesis was performed by using the iScript™ cDNA Synthesis Kit (Bio‐Rad). Quantitative Real‐Time PCR (qRT‐PCR) was performed in a Bio‐Rad CFX96 Real‐Time PCR system and was repeated at least three times. Expression of the housekeeping gene *pmp‐3* was used as an internal control for normalization. For measuring *atgl‐1* mRNA levels we used the primer pairs: 5′‐TGCAAATGCTTTGAACAGCTTC‐3′ and 5′‐CTGGAATACTGAACGTTCTGCAG‐3′ and for measuring *pmp‐3* mRNA levels: 5′‐ ATGATAAATCAGCGTCCCGAC‐3′ and 5′‐ TTGCAACGAGAGCAACTGAAC ‐3′.

### 4.6 Nuclear fractionation

For nuclear fractionation, we followed a previously described protocol (Mata‐Cabana et al., 2018). Briefly, a large number (approximately 250–500 μl worm pellet) of day‐1 adult animals of the wild‐type (N2), and VS20 [*hjIs67*[p_*atgl‐1*_ATGL‐1::GFP;p_*mec‐7*_RFP]] strain were collected from 94 mm plates, respectively. The animals were washed twice with M9 buffer (22 mM KH_2_PO_4_, 42 mM Na_2_HPO_4_, 85.5 mM NaCl, 1 mM MgSO_4_) to remove any remaining bacteria. The worm pellet was washed twice with 1 ml cold hypotonic buffer (15 mM HEPES KOH pH 7.6, 10 mM KCl, 5 mM MgCl_2_, 0.1 mM EDTA, 350 mM sucrose). The hypotonic buffer was then removed and replaced with 0.5 ml fresh hypotonic buffer, supplemented with 1 mM DTT and 2× protease inhibitors (cOmplete mini protease inhibitors cocktail tablets – ROCHE). The worms were homogenized with 100 strokes in a 3 ml Potter‐Elvehjem homogenizer with PTFE pestle and glass tube (Sigma‐Aldrich). The lysates were centrifuged for 5 min at 500 *g* at 4°C for the precipitation of worm debris. The supernatant was transferred to a new tube and centrifuged again for 5 min at 500 *g*, 4°C. Similarly, the resulting supernatant was transferred to a new tube and 30 μl was kept as the input fraction. The nuclei were subsequently pelletized by centrifuging the supernatant at 4000 *g* for 5 min at 4°C. The supernatants were subjected to another round of centrifugation at 17000 *g* for 30 min at 4°C, to produce the cytoplasmic fraction. The pellets were washed twice with hypotonic buffer and centrifuged at 4000 *g* for 5 min at 4°C. Nuclear pellets were finally resuspended in 50 μl hypertonic buffer (15 mM HEPES KOH pH 7.6, 400 mM KCl, 5 mM MgCl_2_, 0.1 mM EDTA, 0.1% Tween‐20, 10% Glycerol, 1 mM DTT, 2× protease inhibitor cocktails) and transferred to a new tube. After snap freezing, all samples were stored at −80°C for further analysis by Western blotting.

### 4.7 BODIPY staining

L4 transgenic larvae expressing p_*app‐1*_H2B::mCherry transgene were placed on nematode growth media *Escherichia coli* (OP50) plates seeded with 100 μl of 5 μM C1BODIPY‐C12 (Invitrogen, D3823) diluted in M9 buffer. 1‐day‐old and 5‐day‐old stained nemaotdes were washed twice with M9 buffer. Then, the animals were immobilized with 10 mM levamisole/M9 buffer before mounting on 2% agarose pads for microscopic examination with a KEYENCE BZ‐X800 epifluorescence microscope. The number of nLDs were calculated for each intestinal nuclei in these images using the ImageJ software (http://rsb.info. nih.gov/ij/). In each experiment, at least 20 animals and intestinal 50 nuclei were examined for each strain/condition. Each assay was repeated at least three times. We used the Prism software package (GraphPad Software) for statistical analyses.

### 4.8 Western blot analysis

Samples from nuclear fractionation were analyzed by SDS‐PAGE (sodium dodecyl sulfate–polyacrylamide gel electrophoresis) and Western blotting. The input, cytoplasmic, and nuclear fractions were boiled for 10 min with 1× Laemmli sample buffer (70 mM SDS, 1.5 mM bromophenol blue, 0.8% glycerol, 10 mM Tris–HCl pH 6.8, 100 mM DTT). The samples were then centrifuged at 17,000 *g* for 5 min at 4°C. The resulting supernatants were loaded onto a 10% SDS‐PAGE gel for separation and then transferred to a nitrocellulose membrane (Amersham‐GE Healthcare). The membrane was blocked in 5% non‐fat milk for 1 h at RT and blotted against GFP (Minotech Biotechnology, 1:10,000), α‐tubulin (DSHB Cat# AA4.3, RRID: AB_579793, 1:10,000), and fibrillarin (Novus Biologicals Cat# NB300‐269, 1:1000) in 5% non‐fat milk at 4°C. After three washes with 1xTris ‐buffered saline ‐Tween‐20 (TBS‐T), the membrane was incubated with a suitable horseradish peroxidase (HRP)‐ secondary antibody for 1 h at RT, and washed again three times with 1XTBS‐T. Finally, the membrane was developed by chemiluminescence (Supersignal chemiluminescent substrate pico and femto, Thermo Fisher Scientific).

## AUTHOR CONTRIBUTIONS

K.P. and M.M. conceived and conceptualized the project. K.P., M.M., and C.P. wrote the paper. K.P., M.M., C.P., and A.P. performed and analyzed the experiments. All authors edited the paper. K.P., G.F., and N.T. supervised the project.

## FUNDING INFORMATION

M.M. is funded by a grant from the Hellenic Foundation for Research and Innovation (HFRI 1357) and the General Secretariat for Research and Technology (GSRT). K.P. is funded by a grant from Fondation Santé. C.P. was supported by the Hellenic Foundation for Research and Innovation (H.F.R.I.) under the “1st Call for H.F.R.I. Research Projects to support Faculty members and Researchers and the procurement of high‐cost research equipment” (Project Number: HFRI‐FM17C3‐0869). N.T. is funded by grants from the European Research Council (ERC – GA695190 – MANNA), the European Commission Framework Programmes, and the Greek Ministry of Education.

## CONFLICT OF INTEREST

The authors declare that there is no conflict of interest.

## Supporting information


Figures S1‐S9

Table S1

## Data Availability

The data that support the findings of this study are included in the main and supplementary figures that accompany the manuscript. The data are also available from the corresponding author upon request.
