# Supplementary material for: Age‐dependent nuclear lipid droplet deposition is a cellular hallmark of aging in Caenorhabditis elegans
Source: Aging Cell. 2023 Jan 31;22(4):e13788. doi: 10.1111/acel.13788 (PMC10086520; doi:10.1111/acel.13788)
Supplement: Supplementary file 1 — Figures S1‐S9 [file ACEL-22-e13788-s002.pdf]

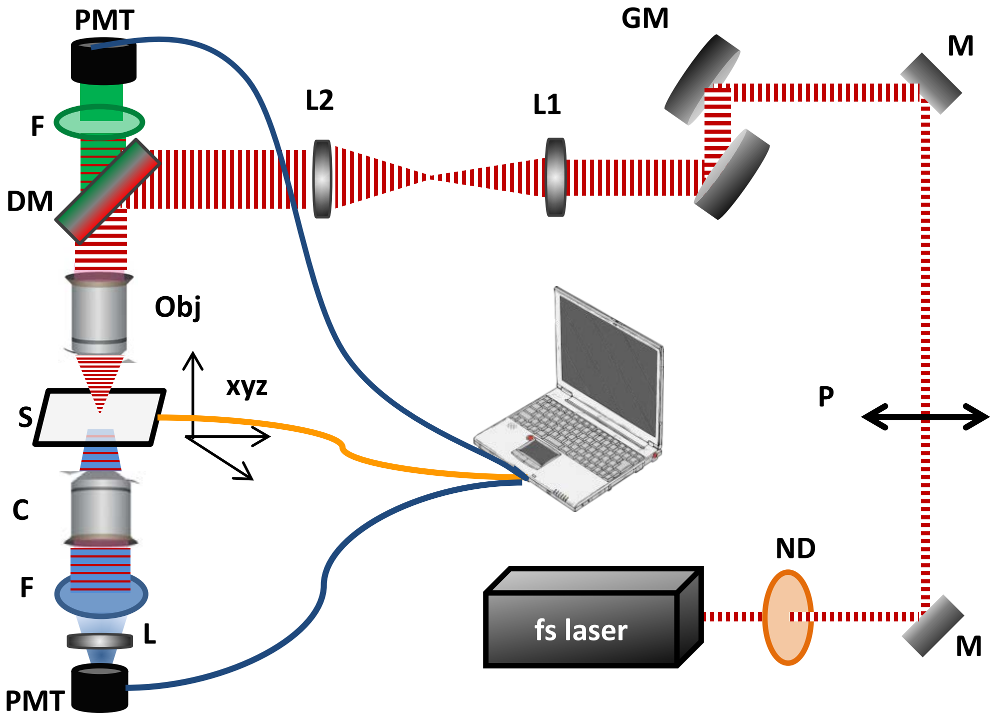

Palikaras et al.
Supplementary Figure 1

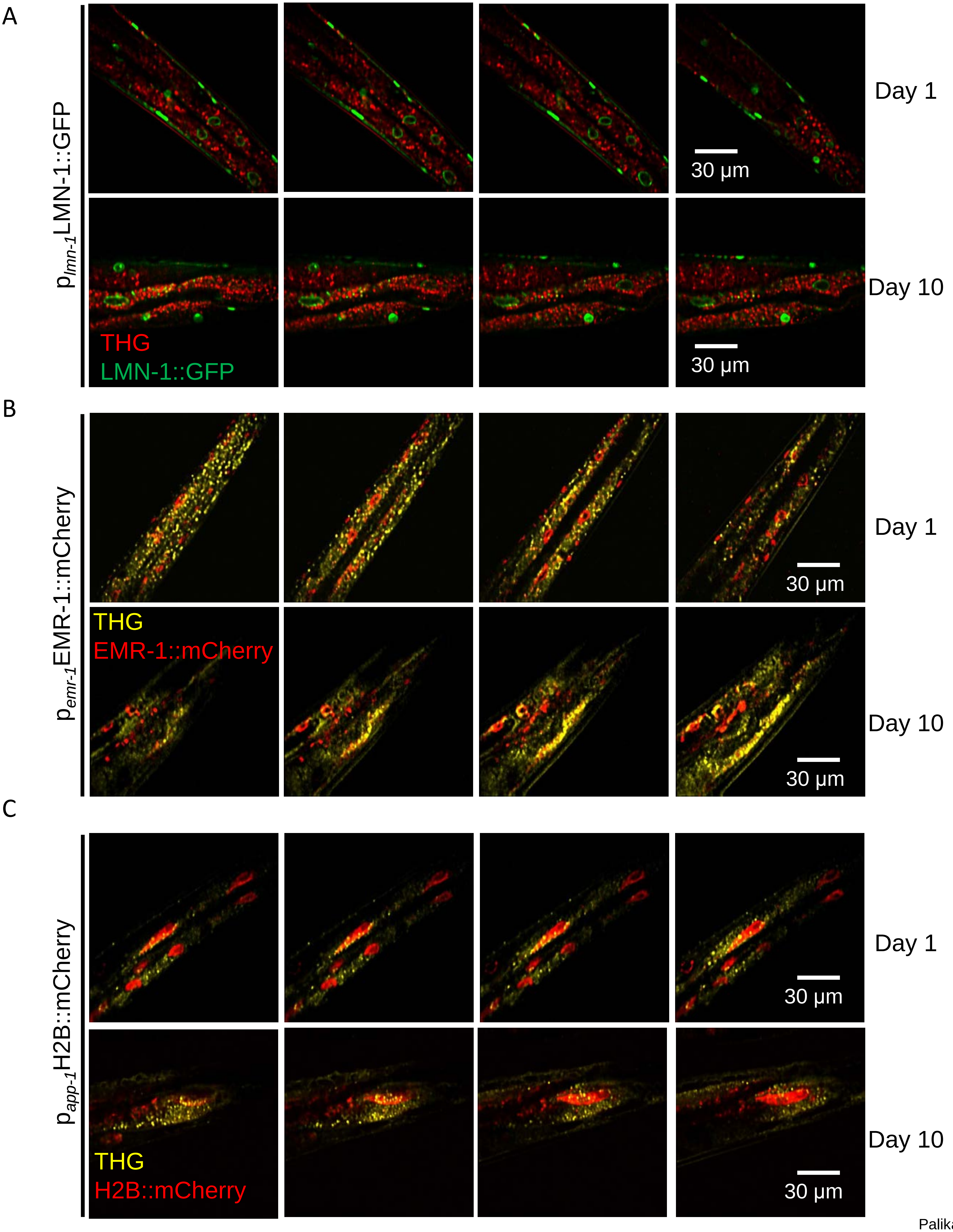

Palikaras et al.
Supplementary Figure 2

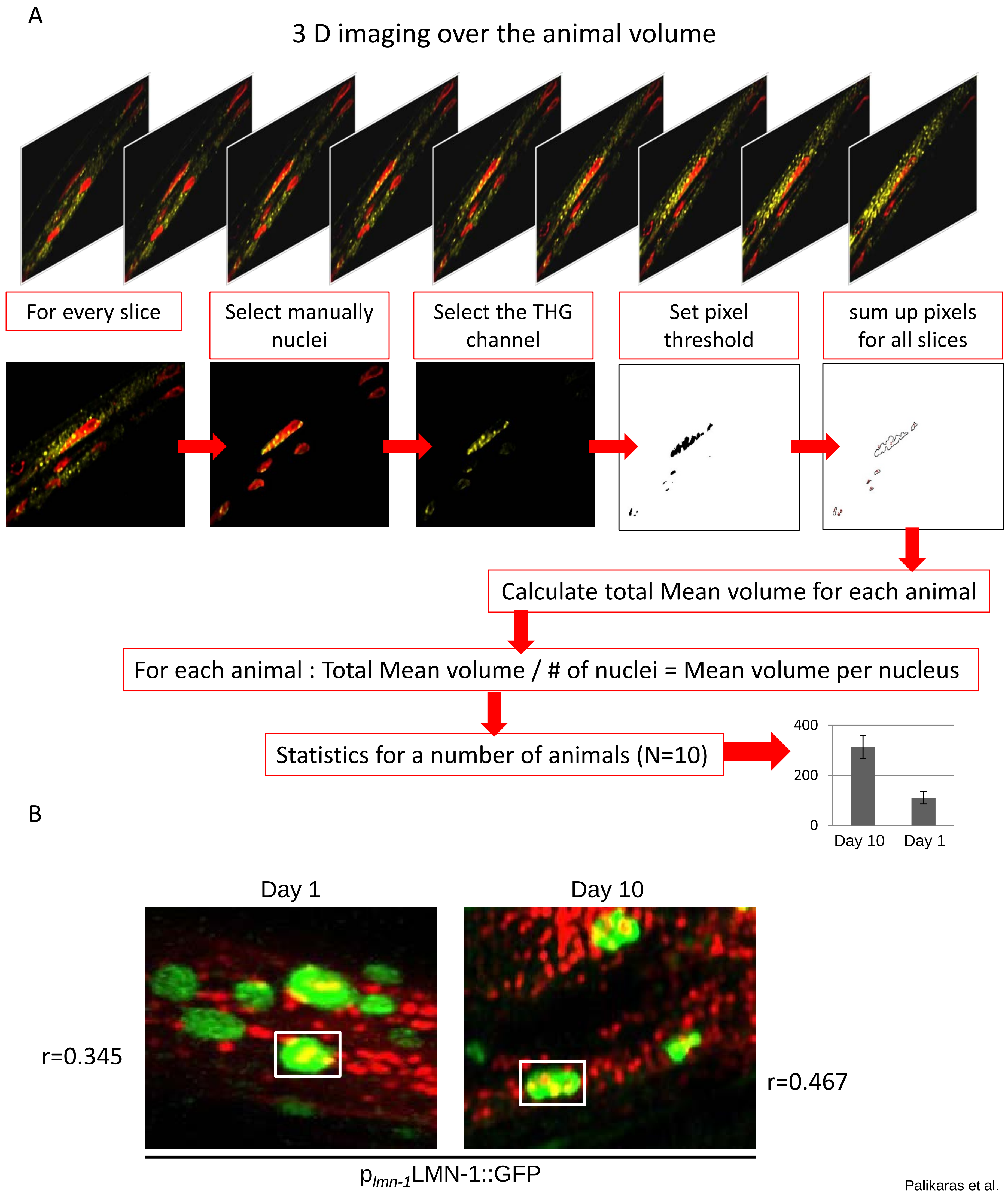

Palikaras et al.
Supplementary Figure 3

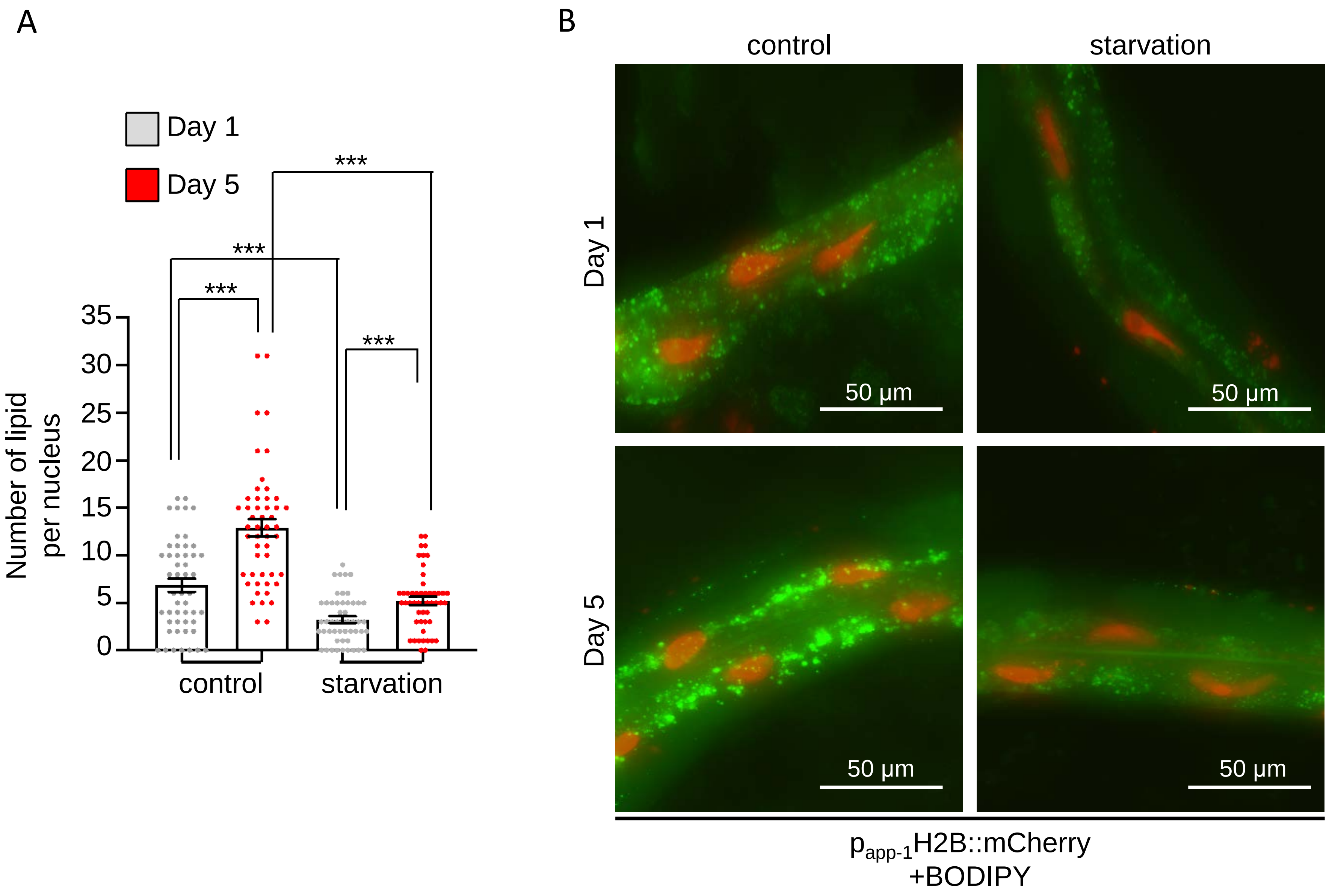
A
Day 1
Day 5
***
***
***
***
Number of lipid per nucleus
35
30
25
20
15
10
5
0
control
starvation
B
control
starvation
Day 1
Day 5
50 μm
50 μm
50 μm
50 μm
p app-1 H2B::mCherry
+BODIPY

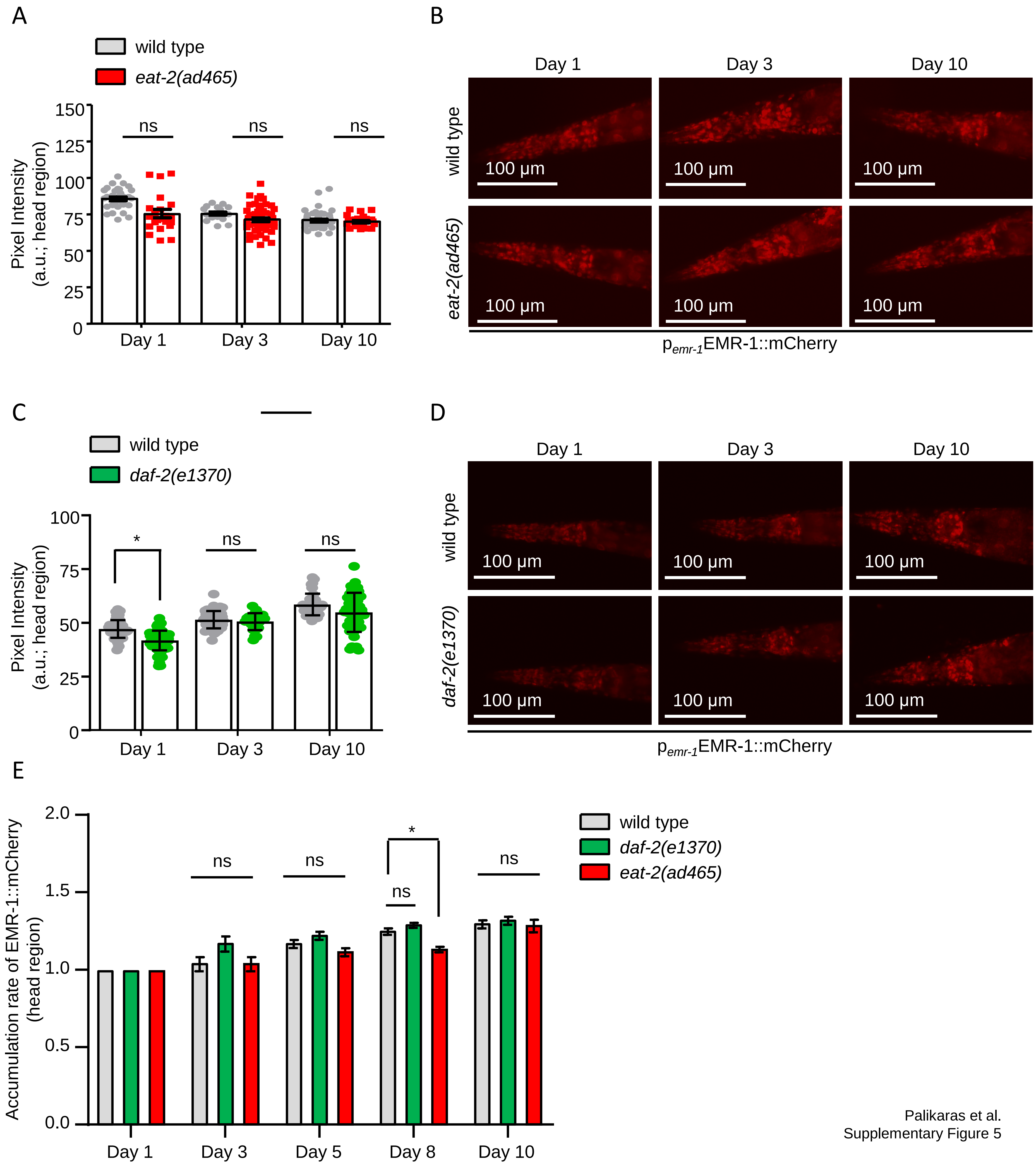

Palikaras et al.
Supplementary Figure 5

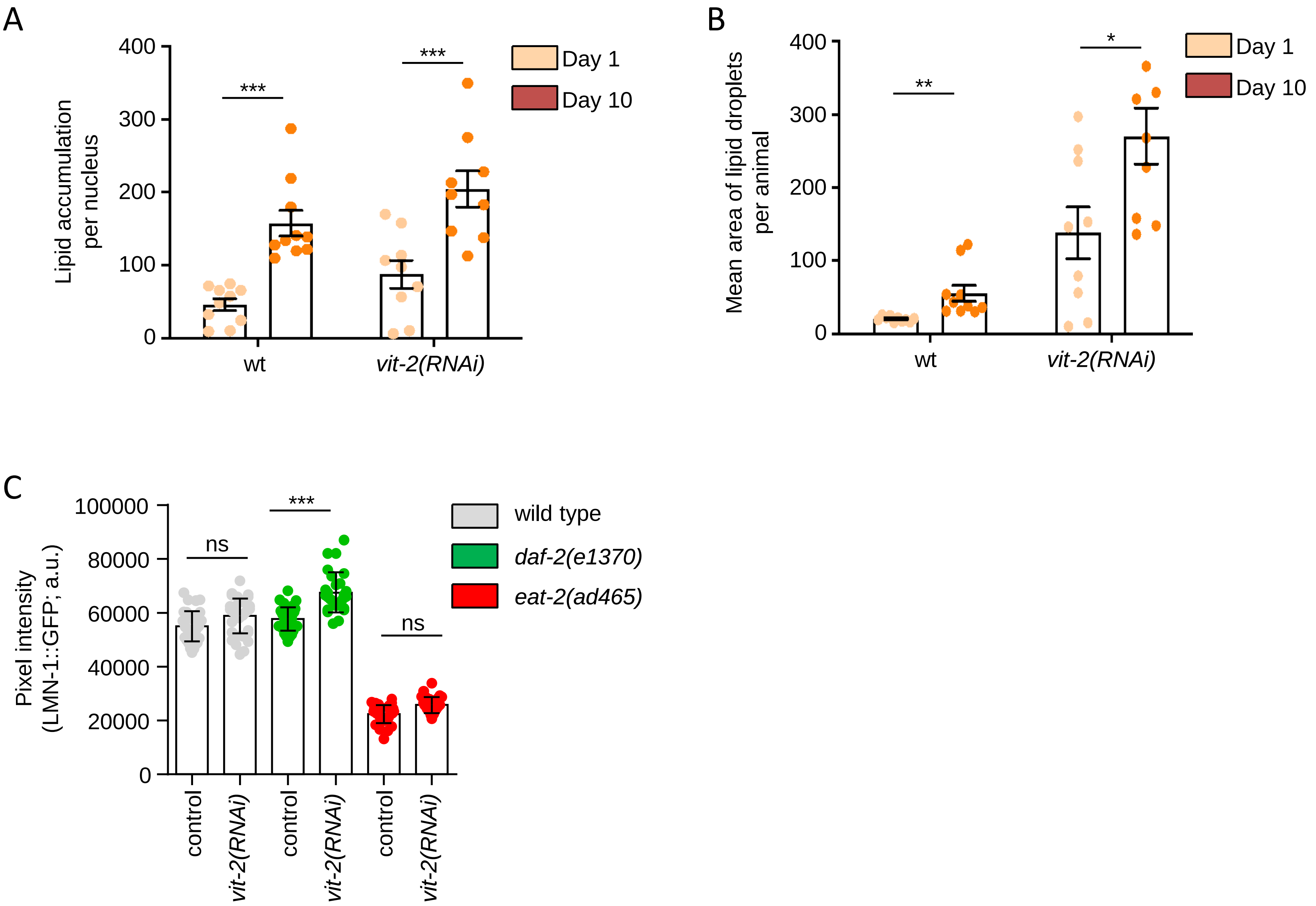

Palikaras et al.
Supplementary Figure 6

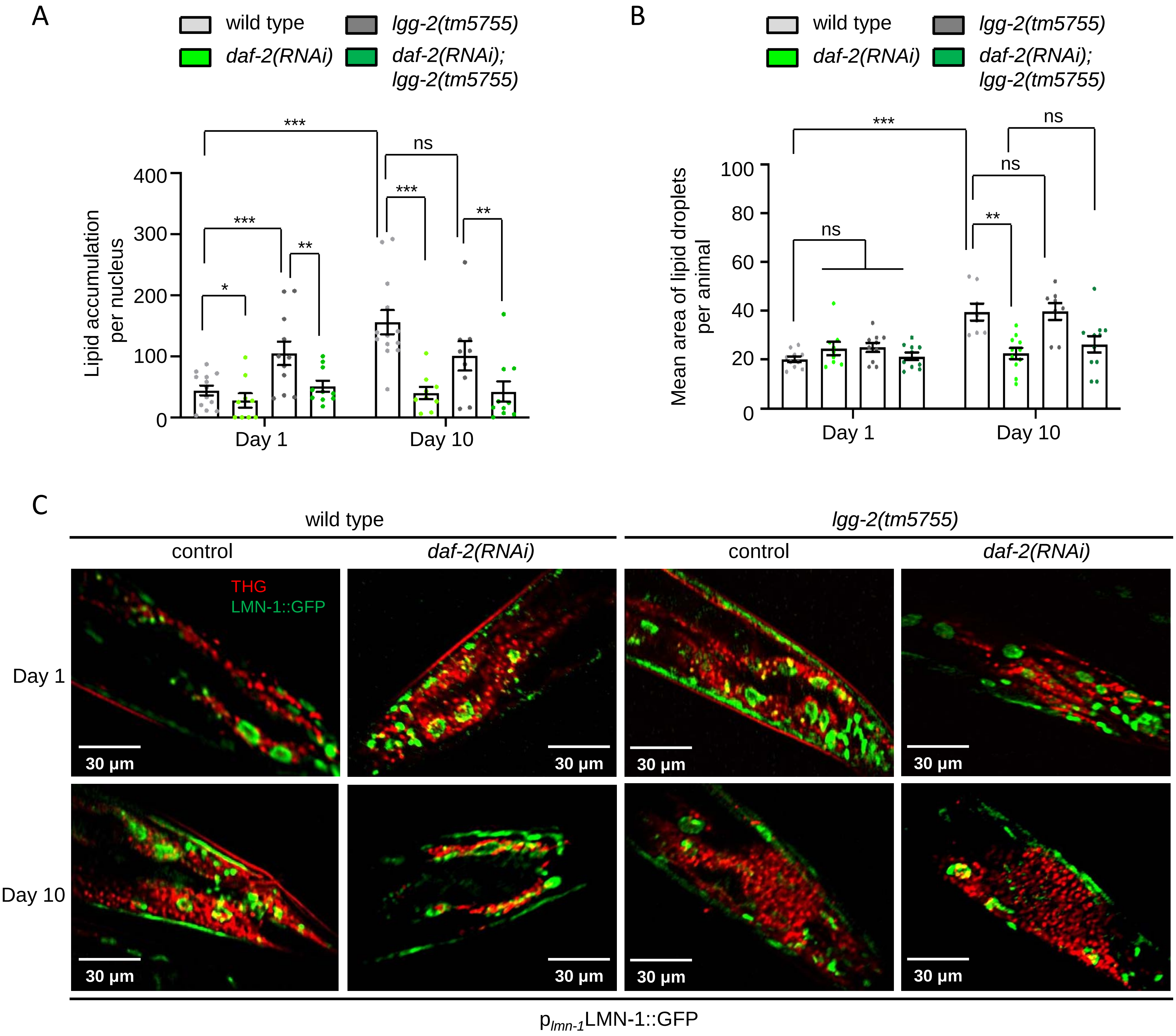

Palikaras et al.
Supplementary Figure 7

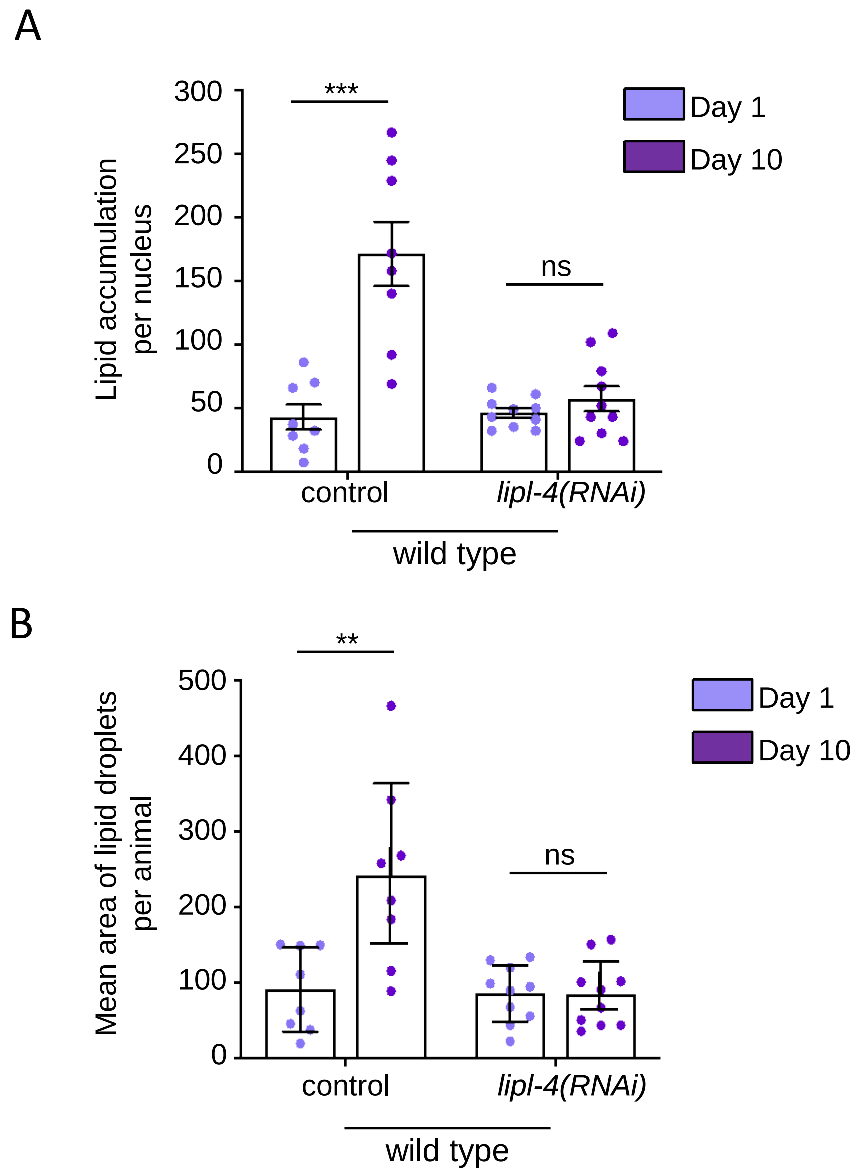

Palikaras et al.
Supplementary Figure 8

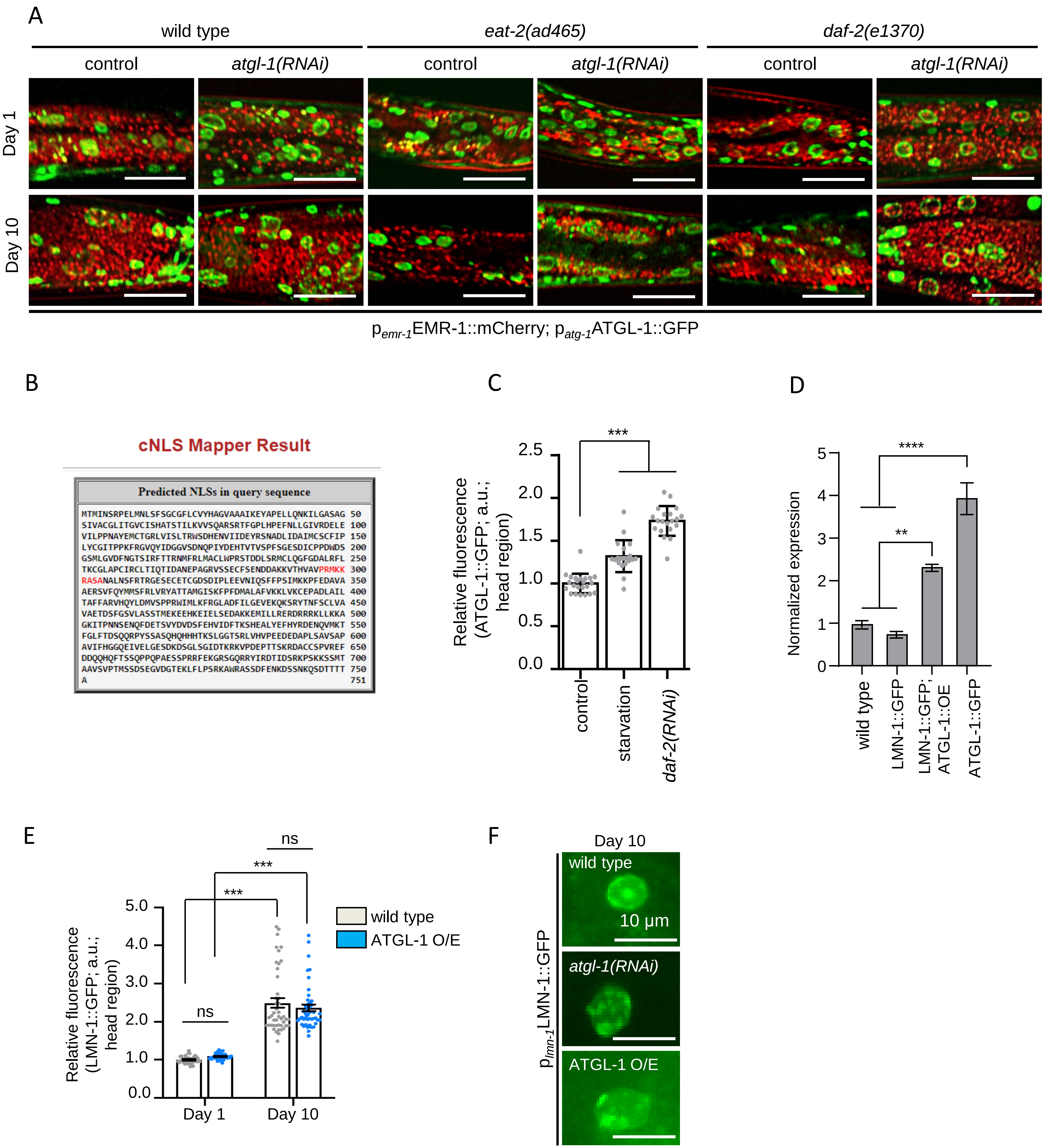

Palikaras et al.
Supplementary Figure 9
