# Supplementary material for: Age‐dependent nuclear lipid droplet deposition is a cellular hallmark of aging in Caenorhabditis elegans
Source: Aging Cell. 2023 Jan 31;22(4):e13788. doi: 10.1111/acel.13788 (PMC10086520; doi:10.1111/acel.13788)
Supplement: Supplementary file 2 — Table S1 [file ACEL-22-e13788-s001.docx]

**Supplementary table 1.** Summary of oligonucleotides used for the generation of expression vectors and RNAi constructs

| **Name** | **Orientation** | **Sequence (5’🡪3’)** | **Final vector** |
| --- | --- | --- | --- |
| *atgl-1* promoter | FW | GATATCAAATCTTAAGCTGGTCCCTATG | p*_atgl-1_*ATGL-1 (with *atgl-1* 3’UTR) in pCRII-TOPO |
| *atgl-1* 3’UTR | RV | CCCGGGAACTTTTTTCAATTTATTTAG |  |
| *atgl-1(RNAi)* | FW | CCGGACGTCTGGTTATCTCG | *atgl-1(RNAi)* in pL4440 |
|  | RV | TCTTCGGCGATCTCGTTCAC |  |
| *bec-1(RNAi)* | FW | GCTCTAGAGTTATCACAGAAGCTCTG | *bec-1(RNAi)* in pL4440 |
|  | RV | CGGGATCCGTCCATACAATGCGTACG |  |
| *daf-2(RNAi)* | FW | CGGGATCCTGTGCCCACGTGGAGCTT | *daf-2(RNAi)* in pL4440 |
|  | RV | CCGCTCGAGTGAATAGCGTCCGAATCG A |  |
| *hlh-30(RNAi)* | FW | TTGACATTTCCAACGAGACGC | *hlh-30(RNAi)* in pL4440 |
|  | RV | TGCTCGTCCCTAGAATTCACA |  |
| *lgg-1(RNAi)* | FW | GGAATTCAAGTGGGCTTACAAGGAG | *lgg-1(RNAi)* in pL4440 |
|  | RV | GGAATTCGTCTTCTTCGTTTATTCATG |  |
| *lgg-2(RNAi)* | FW | GGAAATCGGGGAGGATCTTACA | *lgg-2(RNAi)* in pL4440 |
|  | RV | CAATGTTCAGGGCAGTAAGGA |  |
| *lipl-4(RNAi)* | FW | CTACAACACAACAACAAAAGAT | *lipl-4(RNAi)* in pL4440 |
|  | RV | ATGAGAAAGAAATTACCTGAAC |  |
| *vps-34(RNAi)* | FW | AGGAAATCCAGTGCCGGTGGTT | *vps-34(RNAi)* in pL4440 |
|  | RV | GCCAAATCTCCACTAGCCGCCT |  |
